# Bis[μ-2,5-bis­(pyridin-2-yl)-1,3,4-thia­diazole-κ^4^ *N* ^2^,*N* ^3^:*N* ^4^,*N* ^5^]bis­[(nitrato-κ*O*)silver(I)] tetra­hydrate

**DOI:** 10.1107/S1600536813014578

**Published:** 2013-05-31

**Authors:** Abdelhakim Laachir, Fouad Bentiss, Salaheddine Guesmi, Mohamed Saadi, Lahcen El Ammari

**Affiliations:** a Laboratoire de Chimie de Coordination et d’Analytique (LCCA), Faculté des Sciences, Université Chouaib Doukkali, BP 20, M-24000 El Jadida, Morocco; b Laboratoire de Chimie du Solide Appliquée, Faculté des Sciences, Université Mohammed V-Agdal, Avenue Ibn Battouta, BP 1014, Rabat, Morocco

## Abstract

The self-assembly of an angular 2,5-bis­(pyridin-2-yl)-1,3,4-thia­diazole ligand (*L*) with silver nitrate (AgNO_3_) produced a new dinuclear silver(I) coordination complex, [Ag_2_(C_12_H_8_N_4_S)_2_(NO_3_)_2_]·4H_2_O, which crystallizes with two Ag atoms bridged by two *L* ligands. The Ag atom is surrounded by four N atoms of *L* and by one O from the nitrate anion defining a distorted square pyramid. The atoms comprising the dication are nearly coplanar, with an r.m.s. deviation of 0.1997 Å. Mol­ecules are linked by C—H⋯O and O—H⋯O hydrogen bonds through nitrate anions and water mol­ecules, forming a two-dimensional porous network. The overall structure involves stacking of Ag complex layers along the *b* axis. The cohesion in the three-dimensional architecture is ensured by O⋯Ag inter­actions.

## Related literature

For the synthesis of the ligand, see: Lebrini *et al.* (2005 ▶). For background to coordination polymers, see: Brammer (2004 ▶); Ghosh *et al.* (2004 ▶); Maspoch *et al.* (2004 ▶). For complexes with the same ligand but with other metals and counter-anions, see: Bentiss *et al.* (2012 ▶); Niu *et al.* (2009 ▶).
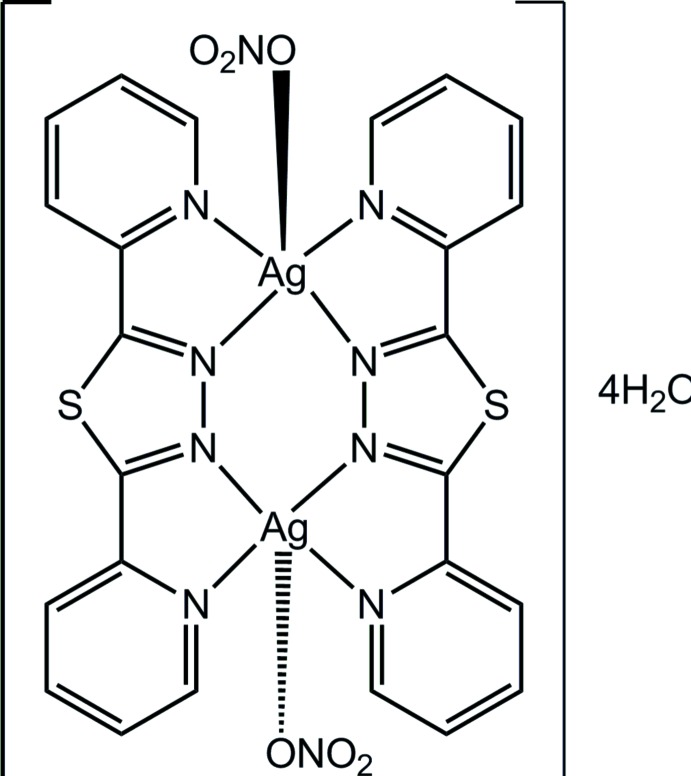


## Experimental

### 

#### Crystal data

[Ag_2_(C_12_H_8_N_4_S)_2_(NO_3_)_2_]·4H_2_O
*M* *_r_* = 892.41
Triclinic, 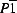

*a* = 5.4251 (1) Å
*b* = 10.6894 (3) Å
*c* = 14.5865 (3) Å
α = 108.910 (1)°
β = 91.447 (1)°
γ = 102.440 (1)°
*V* = 777.30 (3) Å^3^
*Z* = 1
Mo *K*α radiation
μ = 1.47 mm^−1^
*T* = 296 K
0.42 × 0.32 × 0.23 mm

#### Data collection

Bruker X8 APEX diffractometer
Absorption correction: multi-scan (*SADABS*; Sheldrick, 2008 ▶) *T*_min_ = 0.739, *T*_max_ = 0.867
29938 measured reflections
5388 independent reflections
3919 reflections with *I* > 2σ(*I*)
*R*_int_ = 0.029

#### Refinement

*R*[*F* ^2^ > 2σ(*F* ^2^)] = 0.034
*wR*(*F* ^2^) = 0.090
*S* = 1.01
5388 reflections
217 parameters
H-atom parameters constrained
Δρ_max_ = 0.92 e Å^−3^
Δρ_min_ = −0.93 e Å^−3^

### 

Data collection: *APEX2* (Bruker, 2009 ▶); cell refinement: *APEX2*; data reduction: *SAINT* (Bruker, 2009 ▶); program(s) used to solve structure: *SHELXS97* (Sheldrick, 2008 ▶); program(s) used to refine structure: *SHELXL97* (Sheldrick, 2008 ▶); molecular graphics: *ORTEP-3 for Windows* (Farrugia, 2012 ▶) and *Mercury* (Macrae *et al.*, 2008 ▶); software used to prepare material for publication: *PLATON* (Spek, 2009 ▶) and *publCIF* (Westrip, 2010 ▶).

## Supplementary Material

Crystal structure: contains datablock(s) I, global. DOI: 10.1107/S1600536813014578/tk5229sup1.cif

Structure factors: contains datablock(s) I. DOI: 10.1107/S1600536813014578/tk5229Isup2.hkl

Additional supplementary materials:  crystallographic information; 3D view; checkCIF report

## Figures and Tables

**Table 1:** Hydrogen-bond geometry (Å, °)

*D*—H⋯*A*	*D*—H	H⋯*A*	*D*⋯*A*	*D*—H⋯*A*
O4—H4*B*⋯O5	0.86	2.08	2.777 (4)	138
O4—H4*A*⋯O4^i^	0.86	2.51	2.980 (9)	115
C1—H1⋯O2^ii^	0.93	2.44	3.342 (3)	164
C12—H12⋯O2^iii^	0.93	2.48	3.376 (4)	162
O5—H5*A*⋯O2^iv^	0.86	2.05	2.874 (3)	162
O5—H5*A*⋯O1^iv^	0.86	2.46	3.191 (3)	143
O5—H5*B*⋯O1^v^	0.86	1.99	2.851 (3)	176

Symmetry codes: (i) 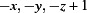; (ii) 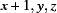; (iii) 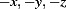; (iv) 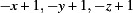; (v) 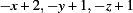.

**Fig. 1.**
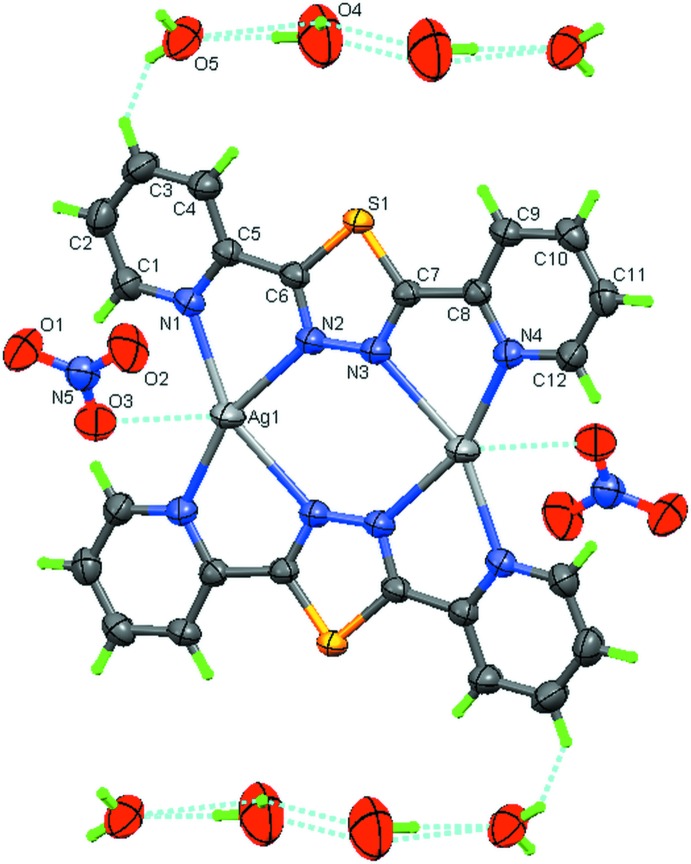
Plot of the crystal structure showing the molecules linked to the silver cation, with the atom-labelling scheme. Displacement ellipsoids are drawn at the 50% probability level. Symmetry code: (i) -x + 1, -y, -z; (ii) -x, -y, -z + 1; (iii) -x + 1, -y + 1, -z + 1.

**Fig. 2.**
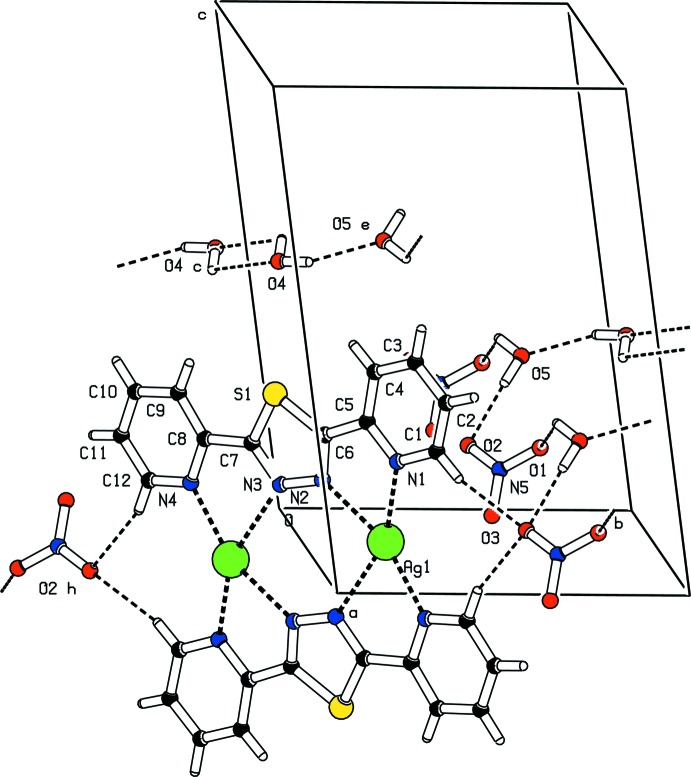
Partial plot of the unit cell showing crystal packing. Hydrogen bonds are depicted as dashed lines. Symmetry codes: (c) -x, -y, -z + 1; (e) -x + 1, -y + 1, -z + 1; (h) -x, -y, -z.

## supplementary crystallographic information

### Comment

The design and construction of novel coordination polymers are very important parts of crystal engineering not only for the purpose of generating functional materials (Maspoch *et al.*, 2004) but also for their fascinating structures (Brammer, 2004). So far, the design and synthesis of new ligands with various coordinating modes, the exploration of synthetic methods to construct coordination polymers, and investigation on the effect of various factors upon architectures have greatly contributed to this field (Ghosh *et al.*, 2004). The compound 2,5-bis(2-pyridyl)-1,3,4-thiadiazole was usually used as a bidentate ligand to form five-atom ring complexes and sometimes it coordinates two metal atoms by four nitrogen atoms as double-bidentate ligand. The structures of monomeric complexes of the neutral 2,5-bis(2-pyridyl)-1,3,4-thiadiazole derivative with Cu^2+^ (nitrate, perchlorate and trifluoromethanesulfonate) and Ag^+^ (SbF_6_^-^) have been previously reported (Bentiss *et al.*, 2012; Niu *et al.*, 2009). Recently, the study of the new di-nuclear silver(I) coordination complexes with 2,5-bis(2-pyridyl)-1,3,4-thiadiazole showed that the supramolecular structures of its silver complexes can change with the size of counter-anions of the same polyhedron (Niu *et al.*, 2009). As a continuation of our work, in this contribution, we report here the synthesis and the single-crystal structure determination of the new dimeric complex formed by 2,5-bis(2-pyridyl)-1,3,4-thiadiazole (*L*) with silver nitrate as counter ions.

The asymmetric unit of the title compound, [2,5-bis(2-pyridyl)-1,3,4- thiadiazole]silver(I) nitrate, dihydrate is doubled by the application of a centre of inversion, resulting in a Ag_2_-containing dimeric complex. The structure shows the silver cation in a distorted square pyramid site formed by four nitrogen atoms belonging to one organic ligand and an O atom of a nitrate anion (Fig. 1). Each ligand molecule is build up by two six-membered rings linked through a five-membered ring. The two silver atoms and ligands are nearly coplanar, with a r.m.s. deviation of 0.1997 Å. The molecules are linked together by C–H···O and O–H···O hydrogen bonds through nitrate and water molecules, forming a two-dimensional porous network. The overall structure involves stacking of Ag complex layers nearly along the *b* axis. The cohesion in the crystal is ensured by O3–Ag1 interaction (Fig. 2 and Table 2).

### Experimental

2,5-Bis(2-pyridyl)-1,3,4-thiadiazole ligand (*L*) was synthesized as described previously by Lebrini *et al.* (2005). AgNO_3_ (0.75 mmol, 0.13 g) in water (5 ml ) was added to *L* (0.21 mmol, 50 mg) dissolved in ethanol (13 ml). The resulting solution was stirred for 30 min. The solution was filtered and allowed to stand at ambient temperature. After seven days, yellow blocks crystallized. Crystals were washed with water and dried under vacuum (yield 34%). These crystals were used as isolated for single-crystal X-ray analysis. Anal. Calc. for C_24_H_24_Ag_2_N_10_O_10_S_2_. C, 37.60; H, 3.13; N, 18.28 S, 8.37; Found: C, 37.69; H, 3.17; N, 18.21; S, 8.34.

### Refinement

H atoms were located in a difference map and treated as riding with C—H = 0.93 Å (aromatic) and O—H = 0.86 Å (water) with *U*_iso_(H) = 1.2 *U*_eq_(aromatic) and *U*_iso_(H) = 1.5 *U*_eq_ (water). The reflections (001), (01–1), (0–11) and (010) are removed from the refinement because they are affected by the beam stop.

### Crystal data

**Table:**

[Ag_2_(C_12_H_8_N_4_S)_2_(NO_3_)_2_]·4H_2_O	*Z* = 1
*M**_r_* = 892.41	*F*(000) = 444
Triclinic, *P*1	*D*_x_ = 1.906 Mg m^−^^3^
Hall symbol: -P 1	Mo *K*α radiation, λ = 0.71073 Å
*a* = 5.4251 (1) Å	Cell parameters from 29938 reflections
*b* = 10.6894 (3) Å	θ = 2.9–32.0°
*c* = 14.5865 (3) Å	µ = 1.47 mm^−^^1^
α = 108.910 (1)°	*T* = 296 K
β = 91.447 (1)°	Block, colourless
γ = 102.440 (1)°	0.42 × 0.32 × 0.23 mm
*V* = 777.30 (3) Å^3^	

### Data collection

**Table:**

Bruker X8 APEX diffractometer	5388 independent reflections
Radiation source: fine-focus sealed tube	3919 reflections with *I* > 2σ(*I*)
Graphite monochromator	*R*_int_ = 0.029
φ and ω scans	θ_max_ = 32.0°, θ_min_ = 2.9°
Absorption correction: multi-scan (*SADABS*; Sheldrick, 2008)	*h* = −8→8
*T*_min_ = 0.739, *T*_max_ = 0.867	*k* = −15→15
29938 measured reflections	*l* = −20→21

### Refinement

**Table:**

Refinement on *F*^2^	Primary atom site location: structure-invariant direct methods
Least-squares matrix: full	Secondary atom site location: difference Fourier map
*R*[*F*^2^ > 2σ(*F*^2^)] = 0.034	Hydrogen site location: inferred from neighbouring sites
*wR*(*F*^2^) = 0.090	H-atom parameters constrained
*S* = 1.01	*w* = 1/[σ^2^(*F*_o_^2^) + (0.0369*P*)^2^ + 0.5008*P*] where *P* = (*F*_o_^2^ + 2*F*_c_^2^)/3
5388 reflections	(Δ/σ)_max_ < 0.001
217 parameters	Δρ_max_ = 0.92 e Å^−^^3^
0 restraints	Δρ_min_ = −0.93 e Å^−^^3^

### Special details

**Table:**

Geometry. All s.u.'s (except the s.u. in the dihedral angle between two l.s. planes) are estimated using the full covariance matrix. The cell s.u.'s are taken into account individually in the estimation of s.u.'s in distances, angles and torsion angles; correlations between s.u.'s in cell parameters are only used when they are defined by crystal symmetry. An approximate (isotropic) treatment of cell s.u.'s is used for estimating s.u.'s involving l.s. planes.
Refinement. Refinement of *F*^2^ against all reflections. The weighted *R*-factor *wR* and goodness of fit *S* are based on *F*^2^, conventional *R*-factors *R* are based on *F*, with *F* set to zero for negative *F*^2^. The threshold expression of *F*^2^ > σ(*F*^2^) is used only for calculating *R*-factors(gt) *etc*. and is not relevant to the choice of reflections for refinement. *R*-factors based on *F*^2^ are statistically about twice as large as those based on *F*, and *R*- factors based on all data will be even larger.

### Fractional atomic coordinates and isotropic or equivalent isotropic displacement parameters (Å^2^)

**Table:**

	*x*	*y*	*z*	*U*_iso_*/*U*_eq_	
Ag1	0.88258 (4)	0.17155 (2)	0.082506 (13)	0.05506 (8)	
S1	0.27700 (10)	−0.00777 (6)	0.27138 (4)	0.03914 (12)	
N1	0.9412 (3)	0.2260 (2)	0.25072 (14)	0.0399 (4)	
N2	0.5007 (4)	0.0657 (2)	0.13988 (14)	0.0442 (4)	
N3	0.2802 (4)	−0.0253 (2)	0.09364 (14)	0.0435 (4)	
N4	−0.1644 (4)	−0.2195 (2)	0.02161 (14)	0.0415 (4)	
C1	1.1468 (4)	0.3134 (3)	0.30566 (19)	0.0473 (5)	
H1	1.2796	0.3477	0.2749	0.057*	
C2	1.1710 (5)	0.3552 (3)	0.4058 (2)	0.0568 (7)	
H2	1.3189	0.4151	0.4414	0.068*	
C3	0.9763 (6)	0.3080 (3)	0.4524 (2)	0.0606 (7)	
H3	0.9894	0.3350	0.5201	0.073*	
C4	0.7590 (5)	0.2191 (3)	0.39710 (17)	0.0491 (6)	
H4	0.6221	0.1863	0.4268	0.059*	
C5	0.7497 (4)	0.1801 (2)	0.29698 (16)	0.0367 (4)	
C6	0.5261 (4)	0.0856 (2)	0.23297 (15)	0.0358 (4)	
C7	0.1435 (4)	−0.0725 (2)	0.15227 (15)	0.0349 (4)	
C8	−0.1038 (4)	−0.1714 (2)	0.11867 (15)	0.0345 (4)	
C9	−0.2607 (4)	−0.2091 (3)	0.18250 (17)	0.0428 (5)	
H9	−0.2114	−0.1748	0.2492	0.051*	
C10	−0.4937 (5)	−0.2991 (3)	0.1457 (2)	0.0476 (5)	
H10	−0.6027	−0.3273	0.1871	0.057*	
C11	−0.5609 (5)	−0.3460 (3)	0.0472 (2)	0.0477 (5)	
H11	−0.7186	−0.4042	0.0210	0.057*	
C12	−0.3919 (5)	−0.3058 (3)	−0.01236 (18)	0.0476 (5)	
H12	−0.4374	−0.3400	−0.0793	0.057*	
O1	1.0220 (4)	0.6251 (2)	0.29332 (16)	0.0703 (6)	
O2	0.6832 (4)	0.4674 (3)	0.2406 (2)	0.0785 (7)	
O3	0.9959 (4)	0.4677 (2)	0.15475 (16)	0.0695 (6)	
N5	0.9004 (4)	0.5189 (2)	0.22918 (16)	0.0474 (5)	
O4	0.2764 (8)	0.0495 (3)	0.5333 (3)	0.1395 (14)	
H4A	0.1511	0.0832	0.5559	0.209*	
H4B	0.4079	0.1163	0.5542	0.209*	
O5	0.4863 (4)	0.3278 (2)	0.61006 (16)	0.0730 (6)	
H5A	0.4020	0.3769	0.6497	0.109*	
H5B	0.6329	0.3453	0.6415	0.109*	

### Atomic displacement parameters (Å^2^)

**Table:**

	*U*^11^	*U*^22^	*U*^33^	*U*^12^	*U*^13^	*U*^23^
Ag1	0.05582 (12)	0.06872 (16)	0.03379 (10)	−0.00435 (9)	0.01077 (7)	0.01998 (9)
S1	0.0410 (3)	0.0453 (3)	0.0288 (2)	0.0034 (2)	0.00920 (19)	0.0134 (2)
N1	0.0394 (9)	0.0424 (11)	0.0376 (10)	0.0057 (8)	0.0089 (7)	0.0152 (8)
N2	0.0441 (10)	0.0516 (12)	0.0328 (9)	−0.0017 (8)	0.0057 (7)	0.0170 (9)
N3	0.0451 (10)	0.0500 (12)	0.0312 (9)	−0.0005 (8)	0.0055 (7)	0.0153 (8)
N4	0.0433 (9)	0.0430 (11)	0.0335 (9)	0.0051 (8)	0.0079 (7)	0.0096 (8)
C1	0.0407 (11)	0.0489 (14)	0.0491 (13)	0.0011 (10)	0.0063 (10)	0.0185 (11)
C2	0.0464 (13)	0.0549 (16)	0.0543 (15)	−0.0029 (11)	−0.0047 (11)	0.0091 (13)
C3	0.0604 (16)	0.073 (2)	0.0359 (13)	0.0036 (14)	−0.0014 (11)	0.0101 (13)
C4	0.0462 (12)	0.0605 (16)	0.0370 (12)	0.0039 (11)	0.0085 (9)	0.0168 (11)
C5	0.0377 (10)	0.0376 (11)	0.0352 (10)	0.0076 (8)	0.0075 (8)	0.0135 (9)
C6	0.0377 (10)	0.0381 (11)	0.0335 (10)	0.0077 (8)	0.0104 (8)	0.0151 (9)
C7	0.0390 (10)	0.0364 (11)	0.0296 (9)	0.0094 (8)	0.0078 (7)	0.0110 (8)
C8	0.0378 (9)	0.0330 (11)	0.0331 (10)	0.0098 (8)	0.0078 (8)	0.0105 (8)
C9	0.0442 (11)	0.0481 (14)	0.0375 (11)	0.0081 (10)	0.0116 (9)	0.0176 (10)
C10	0.0427 (11)	0.0496 (14)	0.0540 (14)	0.0082 (10)	0.0157 (10)	0.0232 (12)
C11	0.0405 (11)	0.0409 (13)	0.0555 (15)	0.0031 (9)	0.0061 (10)	0.0124 (11)
C12	0.0474 (12)	0.0468 (14)	0.0387 (12)	0.0017 (10)	0.0030 (9)	0.0076 (10)
O1	0.0593 (12)	0.0748 (15)	0.0585 (12)	0.0083 (10)	−0.0056 (10)	0.0037 (11)
O2	0.0413 (10)	0.0866 (16)	0.1001 (18)	0.0029 (10)	0.0200 (11)	0.0283 (14)
O3	0.0732 (13)	0.0715 (14)	0.0570 (12)	0.0109 (11)	0.0245 (10)	0.0152 (11)
N5	0.0367 (9)	0.0564 (13)	0.0502 (12)	0.0083 (9)	0.0025 (8)	0.0211 (10)
O4	0.169 (4)	0.077 (2)	0.154 (4)	−0.004 (2)	−0.004 (3)	0.037 (2)
O5	0.0582 (11)	0.0823 (16)	0.0599 (13)	0.0109 (11)	0.0061 (10)	0.0029 (11)

### Geometric parameters (Å, º)

**Table:**

Ag1—N4^i^	2.2785 (19)	C3—H3	0.9300
Ag1—N1	2.3273 (19)	C4—C5	1.379 (3)
Ag1—N2	2.4421 (19)	C4—H4	0.9300
Ag1—N3^i^	2.5519 (19)	C5—C6	1.472 (3)
Ag1—O3	2.911 (2)	C7—C8	1.476 (3)
S1—C6	1.719 (2)	C8—C9	1.374 (3)
S1—C7	1.721 (2)	C9—C10	1.386 (3)
N1—C1	1.334 (3)	C9—H9	0.9300
N1—C5	1.343 (3)	C10—C11	1.369 (4)
N2—C6	1.303 (3)	C10—H10	0.9300
N2—N3	1.365 (3)	C11—C12	1.376 (4)
N3—C7	1.299 (3)	C11—H11	0.9300
N3—Ag1^i^	2.5519 (19)	C12—H12	0.9300
N4—C12	1.342 (3)	O1—N5	1.247 (3)
N4—C8	1.345 (3)	O2—N5	1.229 (3)
N4—Ag1^i^	2.2785 (19)	O3—N5	1.230 (3)
C1—C2	1.376 (4)	O4—H4A	0.8601
C1—H1	0.9300	O4—H4B	0.8601
C2—C3	1.365 (4)	O5—H5A	0.8601
C2—H2	0.9300	O5—H5B	0.8600
C3—C4	1.384 (4)		
			
N4^i^—Ag1—N1	129.5 (1)	C5—C4—H4	120.7
N4^i^—Ag1—N2	159.9 (1)	C3—C4—H4	120.7
N1—Ag1—N2	70.1 (1)	N1—C5—C4	122.9 (2)
N4^i^—Ag1—N3^i^	69.0 (1)	N1—C5—C6	115.1 (2)
N1—Ag1—N3^i^	157.2 (1)	C4—C5—C6	122.0 (2)
N2—Ag1—N3^i^	90.9 (1)	N2—C6—C5	121.8 (2)
N4^i^—Ag1—O3	79.4 (1)	N2—C6—S1	113.6 (2)
N1—Ag1—O3	76.6 (1)	C5—C6—S1	124.6 (2)
N2—Ag1—O3	113.6 (1)	N3—C7—C8	122.4 (2)
N3^i^—Ag1—O3	124.0 (1)	N3—C7—S1	113.7 (2)
C6—S1—C7	87.2 (1)	C8—C7—S1	123.9 (2)
C1—N1—C5	117.4 (2)	N4—C8—C9	123.0 (2)
C1—N1—Ag1	123.0 (2)	N4—C8—C7	114.9 (2)
C5—N1—Ag1	119.2 (2)	C9—C8—C7	122.1 (2)
C6—N2—N3	112.7 (2)	C8—C9—C10	118.7 (2)
C6—N2—Ag1	113.1 (2)	C8—C9—H9	120.6
N3—N2—Ag1	133.5 (2)	C10—C9—H9	120.6
C7—N3—N2	112.8 (2)	C11—C10—C9	118.9 (2)
C7—N3—Ag1^i^	109.7 (2)	C11—C10—H10	120.5
N2—N3—Ag1^i^	135.3 (2)	C9—C10—H10	120.5
C12—N4—C8	117.2 (2)	C10—C11—C12	119.1 (2)
C12—N4—Ag1^i^	120.8 (2)	C10—C11—H11	120.5
C8—N4—Ag1^i^	121.8 (2)	C12—C11—H11	120.5
N1—C1—C2	123.0 (2)	N4—C12—C11	123.0 (2)
N1—C1—H1	118.5	N4—C12—H12	118.5
C2—C1—H1	118.5	C11—C12—H12	118.5
C3—C2—C1	119.4 (2)	N5—O3—Ag1	115.4 (2)
C3—C2—H2	120.3	O2—N5—O3	120.5 (2)
C1—C2—H2	120.3	O2—N5—O1	119.2 (2)
C2—C3—C4	118.7 (3)	O3—N5—O1	120.3 (2)
C2—C3—H3	120.6	H4A—O4—H4B	104.9
C4—C3—H3	120.6	H5A—O5—H5B	104.9
C5—C4—C3	118.6 (2)		
			
N4^i^—Ag1—N1—C1	11.5 (2)	Ag1—N2—C6—S1	−171.2 (1)
N2—Ag1—N1—C1	−173.9 (2)	N1—C5—C6—N2	−10.6 (3)
N3^i^—Ag1—N1—C1	150.9 (2)	C4—C5—C6—N2	168.9 (2)
O3—Ag1—N1—C1	−52.4 (2)	N1—C5—C6—S1	169.5 (2)
N4^i^—Ag1—N1—C5	−176.3 (2)	C4—C5—C6—S1	−11.0 (3)
N2—Ag1—N1—C5	−1.7 (2)	C7—S1—C6—N2	−0.1 (2)
N3^i^—Ag1—N1—C5	−36.8 (3)	C7—S1—C6—C5	179.8 (2)
O3—Ag1—N1—C5	119.8 (2)	N2—N3—C7—C8	179.5 (2)
N4^i^—Ag1—N2—C6	164.0 (2)	Ag1^i^—N3—C7—C8	−14.6 (3)
N1—Ag1—N2—C6	−3.8 (2)	N2—N3—C7—S1	0.0 (3)
N3^i^—Ag1—N2—C6	163.3 (2)	Ag1^i^—N3—C7—S1	165.9 (1)
O3—Ag1—N2—C6	−68.5 (2)	C6—S1—C7—N3	0.1 (2)
N4^i^—Ag1—N2—N3	−4.9 (4)	C6—S1—C7—C8	−179.5 (2)
N1—Ag1—N2—N3	−172.7 (2)	C12—N4—C8—C9	1.4 (3)
N3^i^—Ag1—N2—N3	−5.6 (3)	Ag1^i^—N4—C8—C9	−173.6 (2)
O3—Ag1—N2—N3	122.5 (2)	C12—N4—C8—C7	−177.5 (2)
C6—N2—N3—C7	0.0 (3)	Ag1^i^—N4—C8—C7	7.5 (3)
Ag1—N2—N3—C7	168.9 (2)	N3—C7—C8—N4	6.5 (3)
C6—N2—N3—Ag1^i^	−161.0 (2)	S1—C7—C8—N4	−174.0 (2)
Ag1—N2—N3—Ag1^i^	8.0 (4)	N3—C7—C8—C9	−172.4 (2)
C5—N1—C1—C2	1.4 (4)	S1—C7—C8—C9	7.1 (3)
Ag1—N1—C1—C2	173.8 (2)	N4—C8—C9—C10	−1.0 (4)
N1—C1—C2—C3	−1.2 (4)	C7—C8—C9—C10	177.9 (2)
C1—C2—C3—C4	−0.1 (5)	C8—C9—C10—C11	−0.9 (4)
C2—C3—C4—C5	1.0 (5)	C9—C10—C11—C12	2.1 (4)
C1—N1—C5—C4	−0.4 (3)	C8—N4—C12—C11	−0.1 (4)
Ag1—N1—C5—C4	−173.1 (2)	Ag1^i^—N4—C12—C11	175.0 (2)
C1—N1—C5—C6	179.1 (2)	C10—C11—C12—N4	−1.7 (4)
Ag1—N1—C5—C6	6.4 (3)	N4^i^—Ag1—O3—N5	−174.6 (2)
C3—C4—C5—N1	−0.8 (4)	N1—Ag1—O3—N5	−39.4 (2)
C3—C4—C5—C6	179.8 (2)	N2—Ag1—O3—N5	21.6 (2)
N3—N2—C6—C5	−179.8 (2)	N3^i^—Ag1—O3—N5	129.9 (2)
Ag1—N2—C6—C5	8.9 (3)	Ag1—O3—N5—O2	−39.5 (3)
N3—N2—C6—S1	0.1 (3)	Ag1—O3—N5—O1	141.5 (2)

Symmetry code: (i) −*x*+1, −*y*, −*z*.

### Hydrogen-bond geometry (Å, º)

**Table:**

*D*—H···*A*	*D*—H	H···*A*	*D*···*A*	*D*—H···*A*
O4—H4*B*···O5	0.86	2.08	2.777 (4)	138
O4—H4*A*···O4^ii^	0.86	2.51	2.980 (9)	115
C1—H1···O2^iii^	0.93	2.44	3.342 (3)	164
C12—H12···O2^iv^	0.93	2.48	3.376 (4)	162
O5—H5*A*···O2^v^	0.86	2.05	2.874 (3)	162
O5—H5*A*···O1^v^	0.86	2.46	3.191 (3)	143
O5—H5*B*···O1^vi^	0.86	1.99	2.851 (3)	176

Symmetry codes: (ii) −*x*, −*y*, −*z*+1; (iii) *x*+1, *y*, *z*; (iv) −*x*, −*y*, −*z*; (v) −*x*+1, −*y*+1, −*z*+1; (vi) −*x*+2, −*y*+1, −*z*+1.
